# Healthcare waste management practices and safety indicators in Nigeria

**DOI:** 10.1186/s12889-017-4794-6

**Published:** 2017-09-25

**Authors:** Abayomi Samuel Oyekale, Tolulope Olayemi Oyekale

**Affiliations:** 1 Department of Agricultural Economics and Extension, North-West University Mafikeng Campus, Mmabatho, 2735 South Africa; 2 Institute of Food Security, Environmental Resources and Agricultural Research (IFSERAR), Federal University of Agriculture, Abeokuta, Nigeria

**Keywords:** Healthcare wastes, Safety indicators, Waste management, Service delivery indicator, Nigeria

## Abstract

**Background:**

Adequate management of healthcare waste (HCW) is a prerequisite for efficient delivery of healthcare services. In Nigeria, there are several constraints militating against proper management of HCW. This is raising some environmental concerns among stakeholders in the health sector. In this study, we analyzed the practices of HCW management and determinants of risky/safe indices of HCW disposal.

**Methods:**

The study used the 2013/2014 Service Delivery Indicator (SDI) data that were collected from 2480 healthcare facilities in Nigeria. Descriptive statistics, Principal Component Analysis (PCA) and Ordinary Least Square (OLS) regression were used to analyze the data.

**Results:**

The results showed that 52.20% and 38.21% of the sampled healthcare facilities from Cross River and Bauchi states possessed guidelines for HCW management, respectively. Trainings on management of HCW were attended by 67.18% and 53.19% of the healthcare facilities from Cross River and Imo states, respectively. Also, 32.32% and 29.50% of healthcare facilities from rural and urban areas previously sent some of their staff members for trainings on HCW management, respectively. Sharp and non-sharp HCW were burnt in protected pits in 45.40% and 45.36% of all the sampled healthcare facilities, respectively. Incinerators were reported to be functional in only 2.06% of the total healthcare facilities. In Bauchi and Kebbi states, 23.58% and 21.05% of the healthcare facilities respectively burnt sharp HCW without any protection. Using PCA, computed risky indices for disposal of sharp HCW were highest in Bayelsa state (0.3070) and Kebbi state (0.2172), while indices of risky disposal of non-sharp HCW were highest in Bayelsa state (0.2868) and Osun state (0.2652). The OLS results showed that at 5% level of significance, possession of medical waste disposal guidelines, staff trainings on HCW management, traveling hours from the facilities to local headquarters and being located in rural areas significantly influenced indices of risky/safe medical waste disposal (*p* < 0.05).

**Conclusion:**

The study concluded that there was low compliance with standard HCW management. It was recommended that possession of HCW management guidelines, staff training on HCW disposal and provision of requisite equipment for proper treatment of HCW would promote environmental safety in HCW disposal.

## Background

Solid waste management is one of the major challenges facing many developing countries. Although some institutional mechanisms for addressing waste accumulation and associated health hazards exist, peculiar implementation lapses due to some logistic and restrictive administrative bottlenecks sometimes make them ineffective. In some instances, illegal disposal of solid wastes poses serious environmental problems to the society. Given that the sixth target of the eleventh Sustainable Development Goal (SDG) emphasizes that countries should by “2030, reduce the adverse per capita environmental impact of cities, including by paying special attention to air quality and municipal and other waste management” [1], enhancement of environmental quality can no longer be left to the whims and wishes of few political clans.

In addition to domestic wastes, other waste products with significant environmental impacts are generated from construction and industrial production, agricultural activities and healthcare service delivery. More specifically, healthcare service delivery processes and their underlying activities often culminate into some waste products, which can constitute some environmental and health hazards to the society [2, 3]. It had been noted that in general, about 85% of waste materials from healthcare facilities belongs to the general waste category [3], while the remaining 15% would comprise of highly infectious or toxic radioactive materials [4, 5].

Therefore, health policy makers and professionals have come to a consensus that efficient management of HCW is an integral part of quality healthcare service delivery [6]. African policy makers cannot ignore this, given the urgent need for significant improvement in some major health indicators, in order to reposition the continent for significant economic growth and development. More importantly, the Health Professional Council of South Africa (HPCSA) [6] submitted that effective management of HCW is an integral component of acceptable healthcare professional practice. Therefore, in absence of some standard prescriptive guidelines for their timely disposal, HCW may pose serious health hazards to the society through their associated environmental pollution and their being channels of some diseases and epidemic outbreaks [7, 8].

Furthermore, HCW management is a principal component of healthcare service delivery, which should be carefully evaluated by healthcare service providers. This is to ensure safety of medical personnel and other healthcare workers who are directly or indirectly involved in the whole processes of HCW generation, collection and disposal. It is sad to however realize that many healthcare facilities in Nigeria do not comply with the professional ethics of HCW management, thereby compromising some internationally acceptable standards. Similarly, absence of functioning platforms for monitoring compliance with ethical standards in HCW disposal, ignorance of assigned staff on some safety practices and deliberate violation of prescribed ethical procedures subject the environment to higher risk of pollution from HCW [9].

Generally, HCW of significant environmental hazards could be in the form of sharp objects, discarded human tissues in the course of surgical operations, blood tissues, patients’ vomits, chemical and pharmaceutical materials. Depending on the level of their associated hazard, HCW are meant to be disposed according to some approved international standards. It is perplexing to note that in many instances, HCW are disposed along with domestic wastes into landfills or municipal’s open waste dumpsites [2]. This increases the risk of human contacts with hazardous and highly infectious waste products and exposes the entire population to several form of environmental pollution [4, 10] because some waste collectors could have access to landfill sites and open waste dumpsites. Therefore, the likelihood of human contact with highly infectious HCW increases when domestic wastes are disposed along with HCW. This calls for more research to study activities of healthcare facilities on the management of their waste products, in order to inform some cogent health and environmental policies.

It should be noted that although the Nigerian guidelines on disposal of HCW emphasize proper tracking in the course of waste disposal, little or no effort is put into effective implementation either by the designated monitoring body or the healthcare facilities [9, 11, 12]. Specifically, Longe and Williams [13] found that in four selected healthcare facilities in Lagos state, the responsibilities of medical waste management were contracted to Lagos State Waste Management Authority (LAWMA). However, majority of the healthcare facilities were involved in waste segregation using some form of colour codes. One fundamental problem is inability of some waste management authorities to ensure people’s safety in the course of waste disposal. This often results through failure to provide adequate covers for waste disposal vehicles, thereby resulting in littering and environmental pollution.

This study seeks to add to existing body of knowledge by analyzing healthcare facilities’ practices in HCW management and determinants of risky and safe disposal of HCW in Nigeria. The study is different from several previous studies by using Principal Component Analysis (PCA) to construct indices of risky and safe HCW management and analyzing these across healthcare facilities’ mode of operation, ownership type, rural/urban location and state of location. The study is also justified from the robustness and representativeness of the dataset.

## Methods

### Study area

Nigeria is the most populous country in Africa [14]. Some projections have indicated that the country’s population will increase from 140 million in 2006 [15] to 204 million by 2020 [14]. The country comprises of 36 states and the Federal Capital Territory (FCT). These states are sub-divided into six geopolitical zones which are the North West, North East, North Central, South West, South East and South South. The country is made up of several ethnic groups although the Hausas, the Yorubas and the Igbos are the predominant groups. Currently ranked as the second largest economy in Africa [16], Nigeria’s performance in achieving Millennium Development Goals (MDGs) was not that impressive. Therefore, conscientious efforts are required in making significant progress on the newly set Sustainable Development Goals (SDGs). This cannot be ignored given that Nigeria’s Human Development Indicator (HDI) increased from 0.467 in 2005 to 0.514 in 2014, with the country ranked 152nd among 188 countries [17].

### Data and sampling procedures

This study used the data that were collected for health Service Delivery Indicator (SDI) in Nigeria [18]. The data were collected between July 2013 and January 2014 with well structured questionnaire comprising of four distinct modules. The first module contained information on selected healthcare facility, the second contained information on staff roster, the third was on patients’ case simulations in order to evaluate the knowledge of healthcare service providers, and the fourth contained facility’s profile of expenditures, resources and governance [19]. Data were collected from selected healthcare facilities using multi-stage cluster sampling with recognition of healthcare facilities’ geographic location (rural/urban) and the type. Sampling was implemented with random selection of two states from each of the six geopolitical zones in Nigeria. The selected states were Kebbi and Kaduna from North West, Bauchi and Taraba from North East, Kogi and Niger from North Central, Ekiti and Osun from South West, Anambra and Imo from South East, and Bayelsa and Cross River from South South. However, the sampling proceeded with stratification beginning at the local government areas. In all, a total of 2480 healthcare facilities were sampled. These comprised of 1480 rural healthcare facilities and 1000 urban healthcare facilities.

Table 1 presents the spatial distribution of healthcare facilities that were sampled. It shows that majority of the sampled healthcare facilities were in rural areas in Taraba, Niger, Kogi, Kaduna, Imo, Cross River, Bayelsa and Bauchi states. The states that were sampled in South West zone had majority of their healthcare facilities located in urban centers. The Table also shows the distribution of the sampled healthcare facilities based on their mode of operation. It reveals that Banchi and Kebbi states had the highest proportions being dispensaries with 45.28% and 47.37%, respectively. None of the selected healthcare facilities in Anambra, Bayelsa, Cross River, Ekiti, Kogi and Osun states was classified as dispensaries. Majority of the healthcare facilities that were sampled in Cross River (90.73%), Anambra (88.44%) and Niger (82.69%) states were classified as health centers. The table also shows the distribution of the sampled healthcare facilities based on type of ownership. It reveals that all the selected healthcare facilities from Anambra state were publicly owned. In the remaining states, healthcare facilities that were publicly owned constituted the highest proportions in Bauchi state (98.58%), Ekiti state (97.60%), Niger state (96.63%) and Cross River state (94.15%). However, the proportions of privately owned healthcare facilities were highest in Imo state (22.17%), Kaduna state (17.67%) and Bayelsa state (14.92%).

**Table 1:** Percentage distribution of healthcare facilities’ location, operations’ mode and ownership

	Location		Mode of operation					Ownership type			
States	Rural	Urban	Dispensary	Health Centre	District Hospital	Other	Missing	Public	Private	Missing	All
Anambra	30.65	69.35	0.00	88.44	8.54	0.50	2.51	100.00	0.00	0.00	199
Bauchi	73.58	26.42	45.28	28.77	10.38	1.89	13.68	98.58	1.42	0.00	212
Bayelsa	57.46	42.54	0.00	69.06	13.81	12.71	4.42	84.53	14.92	0.55	181
Cross River	75.12	24.88	0.00	90.73	8.29	0.00	0.98	94.15	5.37	0.49	205
Ekiti	4.81	95.19	0.00	67.31	8.17	20.19	4.33	97.60	1.92	0.48	208
Imo	87.39	12.61	2.61	70.87	8.26	3.91	14.35	77.39	22.17	0.43	230
Kaduna	63.26	36.74	1.40	70.23	13.02	4.19	11.16	81.86	17.67	0.47	215
Kebbi	78.47	21.53	47.37	42.11	8.61	0.00	1.91	98.56	0.48	0.96	209
Kogi	55.83	44.17	0.00	68.45	30.10	0.00	1.46	92.72	6.31	0.97	206
Niger	81.25	18.75	3.37	82.69	6.25	4.33	3.37	96.63	2.40	0.96	208
Osun	21.50	78.50	0.00	78.04	9.35	1.87	10.75	85.98	14.02	0.00	214
Taraba	84.97	15.03	26.42	50.26	8.81	0.52	13.99	90.67	8.29	1.04	193
All	59.68	40.32	10.56	67.22	11.09	4.11	7.02	91.45	8.02	0.52	2480

### Principal component analysis (PCA) indicator computation

Principal Component Analysis (PCA) was used to aggregate some variables into composite indices, which were further subjected to some descriptive and inferential analyses. The use of PCA is justified given its ability to effectively generate some new uncorrelated variable(s) from a set of several highly correlated variables using orthogonal transformation [20]. PCA also affords elimination of multicollinearity in estimated variables given it unique data aggregation ability [21]. In this study, STATA 12 software was used for data analysis. The software is able to invoke “predict” command to generate new variable(s) after invoking the conventional “pca” command. Indices of risky and safe disposal of sharp and non-sharp HCW were computed as new variables. Specifically, risky indices of HCW disposal were computed from the answers that were provided by sampled healthcare facilities to those questions on disposal of HCW through open burning without protection, dumped without burning (no protection), dumped without burning in open pit without protection and removed off-site in unprotected area. However, safe indices of HCW disposal were computed with healthcare facilities’ responses to those questions on disposal of HCW in open burning pit or protected ground, dumped without burning in covered pit or pit latrine, dumped without burning in protected ground or pit, removed off-site and stored in covered containers, removed off-site and stored in other protected environment and removed off-site and burned with incinerators. Data for PCA analysis were presented with yes responses coded as one and no or missing responses coded as zero.

### Ordinary Least Square (OLS) regression

The indices of safe and risky HCW disposal, which were computed with PCA were subjected to OLS regression analysis. Some standard econometric tests were carried out in order to determine the suitability of conventional OLS regression method for the estimated models [22]. More importantly, the independent variables were examined for multicollinearity using Variance Inflation Factor (VIF) [23]. The presence of heteroscedasticity was also examined with Breusch-Pagan/Cook-Weisberg test [24]. For the models where heteroscedasticity test showed statistical significance (*p* ≤ 0.05), the coefficients of the explanatory variables were computed with robust standard error. Given that ***Y***_***ik***_ represents the indices of risky/safe disposal of HCW, the following models were estimated for risky disposal of sharp HCW (***Y***_***i***1_), risky disposal of non-sharp HCW (***Y***_***i***2_), safe disposal of sharp HCW (***Y***_***i***3_) and safe disposal of non-sharp HCW (***Y***_***i***4_):

1 $$ {Y}_{i1}={\pi}_1+{\theta}_s\sum_{s=1}^{15}{Z}_{is}+{h}_i $$

2 $$ {Y}_{i2}={\pi}_2+{\beta}_s\sum_{s=1}^{15}{Z}_{is}+{u}_i $$

3 $$ {Y}_{i3}={\pi}_3+{\mu}_s\sum_{s=1}^{15}{Z}_{is}+{v}_i $$

4 $$ {Y}_{i4}={\pi}_4+{\gamma}_s\sum_{s=1}^{15}{Z}_{is}+{c}_i $$

where *π*_*k*_, *θ*_*s*_, *β*_*s*_ **,** *μ*_*s*_, and *γ*_*s*_ are the vectors of estimated parameters. Also, *Z*_*ik*_ is a vector of the explanatory variables which are staff received training on waste management (yes = 1, 0 otherwise), healthcare facility located in southern states (yes = 1, 0 otherwise), rural health facility (yes = 1, 0 otherwise), public health facility (yes = 1, 0 otherwise), dispensaries/health center (yes = 1, 0 otherwise), traveling hours to headquarters, access to electricity (yes = 1, 0 otherwise), access to generators (yes = 1, 0 otherwise), batteries as second source of power (yes = 1, 0 otherwise), solar panel as second source of power (yes = 1, 0 otherwise), other source of power (yes = 1, 0 otherwise), access to improved water source (yes = 1, 0 otherwise), number of outpatient hours per day and possession of standard waste management guidelines (yes = 1, 0 otherwise). The stochastic error terms are denoted as *h*_*i*_, *u*_*i*_, *v*_*i*_ and *c*_*i*_.

## Results

### Possession of waste management guidelines and staff trainings

Table 2 shows the distribution of the sampled healthcare facilities based on possession of standard guidelines for medical waste management. It shows that healthcare facilities from Cross River state (52.20%) and Bauchi state (38.21%) reported the highest percentages, while the lowest percentages were from Osun state (12.62%) and Ekiti state (15.87%). In addition, urban healthcare facilities had higher proportion (26.20%) possessing standard waste management guidelines, when compared to their rural counterparts (24.39%). Also, public healthcare facilities had higher proportion (25.40%) having standard medical waste management guidelines, when compared to those that were privately owned (21.11%). Based on mode of operation, district hospitals reported highest percentage (40.36%) having waste management guidelines, while healthcare facilities that were classified as “others” reported the lowest percentage (18.63%).

**Table 2:** Distribution of Healthcare Facilities Based on Possession of Medical Waste Management Guidelines and Training of Staff on Waste Management

	Waste Management Guideline		Waste Management Training	
Variables	No	Yes	No	Yes
States				
Anambra	75.88	24.12	74.37	25.63
Bauchi	61.79	38.21	60.85	39.15
Bayelsa	77.35	22.65	71.82	28.18
Cross River	47.80	52.20	32.84	67.16
Ekiti	84.13	15.87	74.04	25.96
Imo	64.35	35.65	46.09	53.91
Kaduna	80.93	19.07	90.70	9.30
Kebbi	77.03	22.97	79.43	20.57
Kogi	82.04	17.96	72.82	27.18
Niger	81.25	18.75	75.00	25.00
Osun	87.38	12.62	70.56	29.44
Taraba	79.79	20.21	79.79	20.21
Sector				
Rural	75.61	24.39	67.68	32.32
Urban	73.80	26.20	70.50	29.50
Ownership Type				
Public	74.60	25.40	68.34	31.66
Private	78.89	21.11	73.23	26.77
Mode of Operation				
Dispensary	79.39	20.61	77.48	22.52
Health Centre	76.06	23.94	67.95	32.05
District Hospital	59.64	40.36	61.82	38.18
Other	81.37	18.63	76.47	23.53

Table 2 also shows the distribution of the healthcare facilities based on attendance of staff members at some trainings on HCW management. It reveals that Cross River state and Imo state reported the highest attendance by staff at trainings on HCW management with 67.18% and 53.19% respectively, while the lowest percentages were reported in Taraba state (20.21%) and Kaduna state (9.30%). However, 32.32% of rural healthcare facilities reported to have sent staff on HCW management trainings, as compared to 29.50% for urban facilities. Attendance of trainings on HCW management was also higher in public healthcare facilities (31.66%) than those that were privately owned (26.77%). In healthcare facilities that were classified as district hospitals and health centers, 38.18% and 32.05% respectively reported to have sent some staff members to HCW management trainings.

### Distribution of HCW management practices

Table 3 shows the distribution of sampled healthcare facilities based on their waste management practices. The results indicated that some healthcare facilities were involved in open burning of sharp and non-sharp HCW wastes in some protected pits. However, in some instances, HCW were burnt without any form of protection. Specifically, the Table shows that healthcare facilities from Niger state reported the lowest practice of open unprotected burning of sharp HCW (6.25%). Other states that reported very low involvement in unprotected open burning of sharp HCW were Taraba (8.29%) and Kogi (8.74%). The states with healthcare facilities that had the highest involvement in burning sharp HCW without protection were Bauchi and Kebbi with 23.58% and 21.05%, respectively. Healthcare facilities from Kogi state (64.08%) and Anambra state (58.29%) reported the highest involvement in burning sharp HCW in protected pits.

**Table 3:** Percentage distribution of waste management practices used by the healthcare facilities

	Anambra	Bauchi	Bayelsa	Cross River	Ekiti	Imo	Kaduna	Kebbi	Kogi	Niger	Osun	Taraba	All
Sharp Disposal													
Open burning no protection	18.59	23.58	19.34	12.68	11.54	10.87	11.16	21.05	8.74	6.25	14.02	8.29	13.79
Open burning pit or protected ground	58.29	47.17	30.39	59.02	45.19	30.43	33.02	41.15	64.08	39.90	49.07	48.19	45.40
Dump without burning no protection	1.51	5.66	3.87	2.93	1.92	0.43	5.58	9.09	4.37	0.48	0.47	2.59	3.23
Dump without burning in covered pit or pit latrine	2.01	8.96	2.76	3.41	1.44	1.74	1.86	9.57	8.25	2.88	3.74	6.22	4.40
Dump without burning Open-pit - no protection	7.04	24.06	1.10	10.73	14.42	4.78	29.77	23.44	8.25	29.81	2.34	22.80	14.96
Dump without burning - Protected ground or pit	18.59	20.28	3.87	17.56	7.69	0.87	6.05	15.79	16.99	16.83	3.74	25.91	12.70
Remove off-site stored in covered container	16.08	58.96	29.28	24.39	12.50	33.04	16.28	10.53	16.02	16.35	22.90	30.57	23.95
Remove off-site stored in other protected environment	1.01	8.96	10.50	2.44	0.96	13.04	0.93	11.96	5.34	3.37	1.87	3.11	5.32
Remove off-site stored unprotected	1.01	6.13	0.55	0.98	0.00	0.43	2.33	2.39	4.37	7.69	0.00	3.11	2.42
Remove off-site other (specify)	4.52	1.42	7.18	6.83	4.81	6.52	1.86	1.91	5.34	0.96	5.14	1.04	3.95
Never has sharp waste	0.50	3.30	1.10	0.00	2.40	0.43	0.00	0.48	0.00	1.44	0.47	0.00	0.85
Remove off-site burn incinerator	0.50	5.19	0.55	1.46	0.48	26.96	0.93	5.74	0.97	0.96	0.93	1.55	4.11
Incinerator 2-chamber	0.00	0.47	0.55	0.98	0.96	0.87	0.93	2.87	1.46	0.96	0.93	0.52	0.97
Incinerator 1-chamber	1.01	2.83	1.66	5.37	6.25	12.17	0.93	4.78	1.94	5.29	0.93	2.07	3.87
HCW Disposal													
Open burning no protection	19.60	25.00	27.07	15.12	17.31	26.96	11.16	21.05	12.14	8.65	28.04	10.88	18.63
Open burning pit or protected ground	55.28	50.94	29.83	58.05	44.23	36.96	33.49	34.93	65.53	39.90	47.20	48.19	45.36
Dump without burning Flat ground - no protection	1.51	8.02	4.42	2.93	2.40	2.17	4.65	9.09	4.37	0.48	1.40	3.63	3.75
Dump without burning covered pit or pit latrine	3.52	11.32	3.31	6.83	3.85	7.83	1.40	8.13	6.80	2.88	4.67	6.22	5.60
Dump without burning Open-pit - no protection	7.04	23.11	2.21	8.78	14.90	5.22	29.30	19.62	7.28	28.85	2.80	22.80	14.40
Dump without burning Protected ground or pit	17.59	20.28	4.42	17.07	6.25	2.17	6.05	12.92	16.99	15.38	1.87	30.05	12.42
Remove off-site stored in covered container	15.58	56.13	20.44	15.61	6.73	14.78	13.02	18.18	12.62	15.87	11.68	24.35	18.71
Remove off-site stored in other protected environment	1.01	7.55	9.94	4.39	1.44	7.83	0.93	12.92	3.88	4.81	2.34	4.15	5.08
Remove off-site stored unprotected	2.01	6.60	1.10	0.00	0.96	0.00	3.72	1.91	3.88	8.17	0.00	2.59	2.58
Remove off-site other (specify)	3.52	1.42	8.29	3.90	3.85	3.48	1.86	1.44	4.37	0.00	3.27	0.52	2.94
Never has medical waste	2.01	0.94	1.10	2.44	2.40	0.43	0.00	0.00	0.00	0.48	0.00	0.00	0.81
Remove off-site burn incinerator	0.50	2.36	0.00	0.98	0.48	7.83	0.47	4.31	1.46	0.48	0.47	1.04	1.77
Disposal incinerator 2-chamber	0.00	2.36	1.10	3.41	3.37	10.00	0.47	4.78	1.46	5.77	0.93	2.07	3.06
Incinerator functional today	0.00	1.89	1.10	2.44	1.92	6.52	0.93	2.39	2.91	2.40	0.47	1.04	2.06
Power source for incinerator available today	2.51	0.94	1.66	0.98	5.29	0.00	1.40	1.44	0.97	0.48	0.47	17.10	2.66

### Indices of risky and safe disposal of sharp and non-sharp HCW

Table 4 presents the results of safe and risky indices of HCW disposal as computed with Principal Component Analysis (PCA). It reveals that across the sampled states, indices of risky disposal of sharp HCW were highest in Bayelsa (0.3070) and Kebbi (0.2172), while it was lowest in Niger (−0.4086) and Taraba (−0.2381). In addition, indices of risky disposal of non-sharp HCW were highest in Bayelsa state (0.2868) and Osun state (0.2652), while they were lowest in Niger state (−0.4511) and Kaduna state (−0.3023). Bauchi state and Imo state had the highest average indices of safe disposal of sharp HCW with 0.6352 and 0.7346, respectively, while Kaduna state and Ekiti state had the lowest average values with −0.3080 and −0.3766, respectively. The results for indices of safe disposal of HCW show that Bauchi state and Kebbi state had the highest values with 0.6769 and 0.3852, respectively, while Osun state and Ekiti state had the lowest values with −0.3486 and −0.3418, respectively.

**Table 4:** Descriptive Statistics of Indices of Safe and Unsafe Disposal of Sharp and Non-Sharp HCW

Variables	Risky Disposal Index - Sharp		Risky Disposal Index – HCW		Safe Disposal Index - Sharp		Safe Disposal Index – HCW	
*States*	Mean	Std Dev	Mean	Std Dev	Mean	Std Dev	Mean	Std Dev
Anambra	0.1392	0.9587	0.0547	0.9145	−0.3539	0.8575	−0.2202	0.8908
Bauchi	0.1892	1.4178	0.1179	1.3550	0.6352	1.2986	0.6769	1.4637
Bayelsa	0.3070	1.0712	0.2868	1.1374	0.0256	0.9500	−0.0707	0.8825
Cross River	0.0128	1.0029	−0.0074	0.9194	−0.1408	1.2494	−0.0689	1.1640
Ekiti	−0.0720	0.9359	−0.0613	1.0031	−0.3766	0.7589	−0.3418	0.7125
Imo	−0.0241	0.7779	0.2345	1.0225	0.7346	1.5517	0.2600	1.4322
Kaduna	−0.1764	1.1798	−0.3023	1.0812	−0.3080	0.7678	−0.3189	0.7225
Kebbi	0.2172	1.4920	0.1317	1.2896	0.1919	1.6565	0.3852	2.1784
Kogi	0.0146	1.0028	−0.0370	0.9942	−0.1621	1.2642	−0.0982	1.2263
Niger	−0.4086	0.8504	−0.4511	0.8723	−0.1395	1.0727	−0.0634	1.0587
Osun	0.0701	0.7732	0.2652	0.9509	−0.2903	0.8983	−0.3486	0.7751
Taraba	−0.2381	1.0200	−0.2349	1.0631	0.1037	1.0291	0.1793	1.0666
*Sectors*								
Rural	−0.0034	1.1337	−0.0029	1.1263	0.0560	1.1956	0.0258	1.1977
Urban	0.0051	0.9872	0.0044	1.0099	−0.0828	1.2218	−0.0383	1.3038
Ownership Types								
Public	−0.0025	1.0885	0.0010	1.0925	−0.0242	1.2120	−0.0266	1.2375
Private	0.0203	0.9212	−0.0216	0.9371	0.2676	1.1501	0.2795	1.2574
*Mode of Operation*								
Dispensary	0.0639	1.2667	0.0039	1.1770	0.0503	0.9586	0.1228	1.1747
Health Centre	−0.0198	1.0486	0.0079	1.0651	−0.0866	1.1958	−0.1241	1.1249
District Hospital	−0.0033	0.9913	−0.0038	1.0642	0.3359	1.4942	0.4703	1.8204
Other	−0.0409	1.0341	−0.2056	0.9475	0.0055	1.0141	0.0447	0.9685

Table 4 also shows that healthcare facilities in rural areas had lower average risky index of sharp HCW disposal with −0.0034, when compared with their counterparts from urban areas with 0.0051. Urban healthcare facilities also had higher index of risky disposal of non-sharp HCW with 0.0044, as compared to −0.0029 for rural healthcare facilities. Similarly, rural healthcare facilities had higher indices of safe disposal of sharp and non-sharp HCW with 0.0560 and 0.0258, respectively. Urban healthcare facilities had average indices of safe disposal of sharp and non-sharp HCW being −0.0828 and −0.0383, respectively. The results revealed that public healthcare facilities reported lower average risky index for disposal of sharp HCW with −0.0025, while private healthcare facilities had lower average index for non-sharp HCW disposal with −0.0216. However, private healthcare facilities reported higher average safe indices for disposal of sharps and non-sharp HCW with 0.2676 and 0.2795, respectively.

Table 4 further shows that based on mode of operation, dispensaries had the highest indices of risky disposal of sharps and non-sharp HCW. In terms of safe HCW management practices, district hospitals reported the highest indices for safe sharp and non-sharp HCW disposal with 0.3359 and 0.4703, respectively. However, facilities that were classified as health centers had the lowest indices for safe sharp and non-sharp HCW disposal with −0.0866 and −0.1241, respectively.

### Determinants of risky and safe indices HCW disposal

Table 5 shows the results of Ordinary Least Square regression of the determinants of risky indices of HCW disposal by healthcare facilities in Nigeria. Multicollinearity among the explanatory variables was not a problem given the very low value of computed average VIF (1.33). The last column of the Table presents the values of tolerance, which all show that multicollinearity was properly addressed as revealed by high tolerance levels. The Breusch-Pagan test statistics for heteroscedasticity were statistically insignificant (*p* > 0.05), implying that heteroscedasticity was not a problem in the estimated models. The computed F-test statistics also show statistical significance (*p* < 0.05), implying that estimated coefficients for the independent variables were not statistically jointly equal to zero. This test also confirms that estimated models produced good fits for the data.

**Table 5:** OLS results of the determinants of risky waste management indices among Nigeria’s healthcare facilities

	Risky Sharp Disposal Index			Risky Medical Waste Disposal Index			Tolerance
Variables	Coef.	Std. Err.	t-stat	Coef.	Std. Err.	t-stat	1/VIF
Staff received training on waste management	−0.0119	0.0505	−0.24	0.0676	0.0504	1.34	85.06
Southern states	0.1851	0.0481	3.85	0.2978	0.0480	6.20	80.39
Rural healthcare facilities	0.0269	0.0490	0.55	0.0592	0.0489	1.21	80.45
Public healthcare facilities	−0.0043	0.0832	−0.05	0.0199	0.0831	0.24	85.84
Dispensaries/health center	−0.0976	0.0602	−1.62	−0.0315	0.0601	−0.52	74.19
Traveling hours to headquarters	0.0057	0.0345	0.17	−0.0157	0.0345	−0.45	85.45
Electricity	−0.0661	0.0558	−1.18	−0.0189	0.0557	−0.34	60.47
Generators	0.0937	0.0630	1.49	0.0439	0.0630	0.70	64.17
Batteries as second power source	−0.1497	0.2574	−0.58	−0.5025	0.2571	−1.95	97.30
Solar panel as second power	0.0425	0.1496	0.28	−0.0659	0.1493	−0.44	94.11
Other source of power	0.0223	0.1648	0.14	−0.0862	0.1646	−0.52	93.97
Improved source of water	−0.1254	0.0469	−2.67	−0.0472	0.0468	−1.01	88.90
Number of outpatient hours per day	−0.0080	0.0034	−2.38	−0.0092	0.0034	−2.73	77.30
Possession of standard waste management guidelines	0.0357	0.0532	0.67	−0.0054	0.0531	−0.10	87.39
Constant	0.1776	0.1365	1.30	0.0290	0.1363	0.21	
F(14,2465)	2.15			3.94			
Prob > F	0.0075			0.0000			
Adj R-squared	0.0065			0.0163			
Breusch-Pagan /Cook-Weisberg	1.28			2.42			
Mean VIF	1.33						

Table 6 presents the results of the regression analyses for the factors explaining safe sharp and non-sharp indices of HCW disposal. The Breusch-Pagan tests for the two models showed statistical significance (*p* < 0.01). This implies that heteroscedasticity was a major problem in the models and there was the need to estimate the parameters with robust standard errors. Therefore, the t-statistics in Table 6 were computed with robust standard errors. The results of F-tests showed that the models were statistically significant (*p* < 0.01), implying that the estimated parameters were not statistically jointly equal to zero.

**Table 6:** Heteroscedasticity Corrected Parameters of Determinants of Safe Waste Management Indices in Nigeria

	Safe Sharp Disposal Index			Safe Non-Sharp Waste Disposal Index			Tolerance
Variables	Coefficients	Robust Std. Error.	t-stat	Coefficients	Robust Std. Error.	t-stat	1/VIF
Staff received training on waste management	0.0870	0.0578	1.51	0.1159	0.0576	2.01	85.06
Southern states	−0.1408	0.0582	−2.42	−0.3251	0.0609	−5.34	80.39
Rural healthcare facilities	0.2264	0.0560	4.04	0.0845	0.0563	1.50	80.45
Public healthcare facilities	−0.1510	0.0860	−1.76	−0.1581	0.0948	−1.67	85.84
Dispensaries/health center	−0.2095	0.0658	−3.18	−0.2706	0.0689	−3.93	74.19
Traveling hours to headquarters	−0.0706	0.0324	−2.18	0.0104	0.0334	0.31	85.45
Electricity	0.0833	0.0563	1.48	0.0595	0.0538	1.11	60.47
Generators	0.1357	0.0723	1.88	0.1577	0.0728	2.17	64.17
Batteries as second power source	0.3293	0.3032	1.09	0.1991	0.3066	0.65	97.30
Solar panel as second power	0.3885	0.2601	1.49	0.6420	0.3006	2.14	94.11
Other source of power	0.0122	0.1500	0.08	0.0116	0.1719	0.07	93.97
Improved source of water	0.0283	0.0474	0.60	0.0352	0.0466	0.76	88.90
Number of outpatient hours per day	−0.0015	0.0036	−0.41	0.0024	0.0035	0.67	77.30
Waste management guidelines	0.3281	0.0661	4.96	0.3844	0.0681	5.65	87.39
Constant	0.2095	0.1395	1.50	0.1615	0.1469	1.10	
F(14,2465)	8.01			8.28			
Prob > F	0.0000			0.0000			
Adj R-squared	0.0488			0.0686			
Breusch-Pagan /Cook-Weisberg	187.35 ***			553.06 ***			
Mean VIF	1.33						

*** - statistically significant at 1% level

In Table 5, the parameters of southern states show statistical significance (*p* < 0.01) in the two models. This implies that holding other variables constant, states in the southern part of Nigeria had their indices of risky sharp and non-sharp HCW disposal increased by 0.1851 and 0.2978 respectively, when compared with their counterparts in northern Nigeria. Similarly in Table 6, the parameters of southern states show statistical significance in the two models (*p* < 0.05). Specifically, healthcare facilities that were located in southern states of Nigeria had their indices of safe sharp and non-sharp HCW disposal reduced by 0.1408 and 0.3251 respectively when compared with their counterparts in northern Nigeria.

Table 5 also shows that healthcare facilities that were using batteries as secondary source of power had their index of risky non-sharp HCW disposal being significantly lower (*p* < 0.10) by 0.5025 when compared with facilities without access to battery energy source. Similarly, in Table 6, healthcare facilities with access to generator had indices of safe sharp and non-sharp HCW disposal being significantly higher (*p* < 0.10) by 0.1357 and 0.1577 respectively when compared to those without access to generators. The parameter of access to solar panel as second source of power shows statistical significance in the safe non-sharp HCW disposal model. This reveals that compared with those without access, healthcare facilities with access to solar power had their safe non-sharp HCW disposal indices increased by 0.3006.

Table 5 shows that the healthcare facilities with access to improved source of drinking water had their risky sharp HCW disposal indices being significantly reduced by 0.1254 when compared with those without access. The parameters of number of outpatient were statistically significant (*p* < 0.05) in the two estimated models. These imply that as outpatient hours increased by one unit, the indices of risky sharp and non-sharp HCW disposal decreased by 0.0080 and 0.0092, respectively.

In Table 6, the parameter of staff attendance of training on HCW management shows statistical significance (*p* < 0.05) for the safe indices of non-sharp HCW disposal. This implies that healthcare facilities that reported training of some staff on HCW management had their safe non-sharp HCW disposal indices being higher by 0.1159, when compared to those healthcare facilities that did not send staff members for such trainings. Also, rural area parameter in the safe sharp HCW disposal model shows statistical significance (*p* < 0.01). This implies that healthcare facilities that were situated in rural areas had their indices of safe sharp disposal being higher by 0.2264, when compared with those in urban areas. The parameters of public healthcare facilities in the two models are statistically significant (*p* < 0.10). The results show that public healthcare facilities had safe sharp and non-sharp HCW disposal indices that were lower by 0.1510 and 0.1581 respectively when compared to those that were owned by private individuals. Similarly, the parameters of dispensaries or health centers show statistical significance (*p* < 0.01). These results show that indices of safe sharp and non-sharp HCW management reduced by 0.2095 and 0.2706 for healthcare facilities that were classified as dispensaries or health centers when compared with other healthcare facilities.

In Table 6, the parameter of traveling hours to headquarters shows statistical significance (*p* < 0.05) in the model for safe sharp HCW disposal. This reveals that if travelling hour to local headquarters increases by one unit, indices of safe sharp HCW disposal will reduce by 0.0706. Finally, Table 6 shows that the parameters of possession of standard non-sharp HCW management guideline show statistical significance (*p* < 0.01) in the two models. These results show that indices of safe sharp and non-sharp HCW disposal increased by 0.3281 and 0.3844 respectively for healthcare facilities that had standard HCW management guidelines, when compared with those that did not have.

## Discussion

The need for proper management of HCW as a means of safeguarding health workers’ safety and preventing undue outbreak of diseases cannot be overemphasized [25, 26]. This study found that majority of the sampled healthcare facilities did not have medical waste disposal guidelines. This is a reflection of poor attitudes by healthcare service providers to the management of their HCW. Several previous studies have indicated unethical conducts by healthcare facilities in their waste disposal activities [27–31]. Many researchers have similarly decried poor coordination and persistent dormancy of institutional frameworks for the management of HCW in Nigeria [32–34]. However, healthcare facilities are able to dismiss their duty of ensuring stringent diligence in HCW management and go scot-free as a result of inability of existing legal provisions to impose a mandatory “duty of care” on healthcare service providers and enforce some stringent penalties to serve as deterrent for defaulters [9]. Abah and Ohimain [35] reported that in a survey of a tertiary health facility in Nigeria, it was found that the hospital did not have waste management manuals. Possession of guidelines on HCW management was found to enhance safe indices of HCW disposal. This is expected since possession of HCW management guideline will assist designated staff members to understand the procedures for treating different components of waste products that are emanating from healthcare centers.

The results also indicated low training of staff on medical waste management. This is a pointer to inadequate attention being given to management of HCW by many healthcare facilities in Nigeria. This situation may be a perfect reflection of inadequacy of available funds which is a major problem confronting many healthcare facilities in Nigeria [36]. Precisely, in a situation where doctors are unable to procure basic medical equipment, funding may not be available for training those who manage HCW. Although the essentiality of trainings cannot be overemphasized in healthcare waste management, some previous studies have highlighted non-compliance on the part of designated institutions and other stakeholders [37].

Awodele et al. [8] found that in some selected healthcare facilities in Lagos, ability of hospital staff with substantial level of training to properly handle HCW was quite better than those with little or no training. Abah and Ohimain [35] also reported that in a tertiary hospital in Nigeria, only 11.5% of the respondents received some trainings that were related to management of HCW, while 46% understood the importance of having in place efficient waste management guidelines. Ogbonna et al. [38] found that in some healthcare facilities in Port Harcourt, inadequate training of designated staff was identified as a major constraint to proper management of HCW.

Other similar studies include Babatola [39], who conducted a study to analyze waste management practices of healthcare facilities in Akure city of Ondo state, Nigeria. Using a sample of twenty healthcare centers, it was found that only 2% of the staff that were handling HCW had undergone some form of trainings in HCW management. Oli et al. [40] also analyzed HCW management practices of healthcare facilities in Southeast Nigeria. The results showed that there was no significant difference in private and public healthcare participants’ knowledge about the risks posed by HCW. In addition, 7.0% and 16.2% of staff from private and public healthcare facilities respectively had previously attended some trainings on HCW disposal, while only 22.12% and 41.82% indicated that requisite logistics and materials for ensuring safety in HCW disposal were always available.

The results show that open burning of sharp HCW and other non-sharp HCW was reported by some healthcare facilities. It was also indicated that some healthcare facilities were burning sharp and HCW in protected pits. Generally, burning of HCW can constitute some form of air pollution as a result of release of some toxic substances into the atmosphere. Given this, drastic reduction in human exposure would still constitute some health hazards even at extremely low doses [41]. Similarly, HPCSA [6] noted that subjecting HCW to burning instead of incinerated will release some pollutants into the atmosphere, as a result of dioxins formation. In absence of burning, some health centers were disposing their wastes in open general places of dumping domestic wastes, while others dump them inside pits. Although land filling of HCW may be considered safe if properly done, contamination of ground water is equally possible in some exceptional cases [42].

Incinerators were used by few healthcare facilities, although the standard for protecting the environment from pollution thereof should be ensured. This is as a result of the likelihood of ashes from incinerating containing some pollutant because they could contain mercury and cadmium [43]. In some previous studies, it was reported that only 30% of the healthcare facilities in Akure were involved in waste segregation and majority were not sterilizing infectious wastes or using incinerators or autoclaves [39]. Similarly, Yelebe et al. [44] analyzed waste disposal behaviour of some healthcare facilities in Bayelsa state of Nigeria. It was found that majority of the healthcare facilities were grossly lacking in adoption of standard HCW management with absence of incinerators and any treatment of wastes before disposal. It was further noted that some healthcare facilities and municipal waste management authorities were burning waste in open pits, thereby compromising human safety as a result of associated environmental pollution. In a recent study, it was found that 1.98% of the healthcare facilities sampled in Ebonyi state followed standard procedures in medical waste management [45].

Healthcare facilities in Bayelsa state had high indices of risky disposal of sharps and ono-sharp HCW. Similarly, Ekiti and Osun states, both from south western part of Nigeria had lowest indices for safe disposal of sharps and non-sharp HCW disposal. The results for Bayelsa state are in line with the findings of Yelebe et al. [44]. Poor management of sharp HCW poses significant health risks to healthcare workers and those in charge of waste disposal. Precisely, World Health Organization [3, 46] indicated that if a person is injured by an injection needle in the course of disposing HCW, the likelihood of being infected with Hepatitis B virus, Hepatitis C virus and HIV are 30%, 1.8% and 0.3% respectively. However, the implication of such injury becomes more alarming given that most of the healthcare facilities do not have insurance coverage for their staff, while functioning mechanisms for compensating victims of occupational health hazards may be completely absent [45].

The results indicated that healthcare facilities in northern Nigeria were performing better in HCW management than their counterparts in the southern parts of the country. One fundamental issue is that for efficient management of HCW, sufficient land space, equipment and well trained personnel cannot be compromised. The spate of urbanization, especially in southern Nigeria may constitute significant land constraint for treating HCW. More importantly, it should also be noted that majority of the sampled healthcare facilities in northern Nigeria were in rural areas. The volume of wastes that would be generated from many of such rural healthcare facilities would be low, thereby facilitating their safe management.

In addition, regular supply of electricity is required for processing HCW where incinerators are used for highly infectious wastes. The problem of power outage is a major issue in healthcare service delivery in Nigeria [47, 48]. Availability of alternative sources of electricity through generator or batteries can facilitate healthcare service delivery. Waste disposal could as well become safer because healthcare facilities with such power sources may as well be able to procure necessary facilities for safe and effective handling of wastes. The results also indicated that access to safe drinking water reduced risky sharp disposal indices. This may also emphasize the role of regular supply of water in the whole process of HCW disposal. Similarly, the longer the time that healthcare facilities are opened for public use, the lower their indices of risky sharp and non-sharp HCW disposal. This may be directly linked to the size of the hospitals in terms of staff and available facilities for ensuring efficient healthcare service delivery. The need for staff training on handling of HCW was reemphasized by the results. This had been previously emphasized in several public health literature [6, 49].

## Conclusion

This study has provided some empirical analyses of factors explaining risky and safe disposal of HCW in Nigeria. The study has benefited from robust dataset that cuts across the six geopolitical zones in Nigeria. One major limitation of the study is inability to probe into different composition of HCW that were generated and their respective quantities due to data limitations. It can however be concluded that healthcare wastes are important component of healthcare service delivery. Without proper disposal, the healthcare system may become a source of environmental pollution, which could degenerate into disease outbreaks. In the light of the findings from this study, some recommendations are hereby made. First, there is the need for proper enforcement of medical guidelines in relation to safety and efficient service delivery. The current situation portends a perplexing neglect of responsibilities and failure of institutional mechanisms that are in place to ensure adequacy of medical service delivery and safety of healthcare workers. One of the fundamental requisites for managing HCW is training of staff members that are directly connected to the whole processes of waste sorting and disposal. This is also emphasizing the need for adequate budgeting in relation to training of staff on appropriate management of HCW. There should also be appropriate channels for educating hospital staff on the acceptable ways of handling and disposing HCW. These may include the use of posters and other audio visual materials. Precisely, adequate awareness should be created on associated health hazards to people as a result of unsafe disposal of HCW. The onerous task of handling HCW requires adequate logistic supports and provision of some essential equipment. In the light of declining funding to healthcare facilities in the public sector, the ordeal of inadequate disposal of HCW would further complicate the whole processes of economic development.

The results also highlight some significant differences between rural/urban and northern/ southern states’ healthcare facilities in handling of HCW. There is therefore the need for creation of more awareness and devotion of more resources to the management of HCW among healthcare facilities in southern Nigeria. Due to high volume of HCW that are daily generated, urban healthcare facilities and public healthcare facilities must have workable and sustainable means of managing their large spectrum of wastes in a way that ensures utmost environmental and human safety. Finally, ensuring adequate supply of power and water is critical for HCW management in Nigeria. Specifically, incinerators cannot be functionally utilized if electricity supply is erratic. Similarly, several processes that are associated with waste disposal would require regular supply of water.

## Abbreviations

HCW: Healthcare Waste
HDI: Human Development Indicator
HIV: Human Immuno-Deficiency Virus
HPCSA: Health Professional Council of South Africa
IHSN: International Household Survey Network
LAWMA: Lagos State Waste Management Authority
MDGs: Millennium Development Goals
OLS: Ordinary Least Square
PCA: Principal Component Analysis
SDG: Sustainable Development Goal
SDI: Service Delivery Indicator
UNDP: United Nations Development Programme
VIF: Variance inflation factor
